# Flavaglines Stimulate Transient Receptor Potential Melastatin Type 6 (TRPM6) Channel Activity

**DOI:** 10.1371/journal.pone.0119028

**Published:** 2015-03-16

**Authors:** Maxime G. Blanchard, Jeroen H. F. de Baaij, Sjoerd A. J. Verkaart, Anke L. Lameris, Christine Basmadjian, Qian Zhao, Laurent Désaubry, René J. M. Bindels, Joost G. J. Hoenderop

**Affiliations:** 1 Department of Physiology, Radboud Institute for Molecular Life Sciences, Radboud University Medical Center, Nijmegen, The Netherlands; 2 Laboratory of Therapeutic Innovation (UMR7200), CNRS-University of Strasbourg, Faculty of Pharmacy, Illkirch, France; University of Houston, UNITED STATES

## Abstract

Magnesium (Mg^2+^) is essential for enzymatic activity, brain function and muscle contraction. Blood Mg^2+^ concentrations are tightly regulated between 0.7 and 1.1 mM by Mg^2+^ (re)absorption in kidney and intestine. The apical entry of Mg^2+^ in (re)absorbing epithelial cells is mediated by the *transient receptor potential melastatin type 6* (TRPM6) ion channel. Here, flavaglines are described as a novel class of stimulatory compounds for TRPM6 activity. Flavaglines are a group of natural and synthetic compounds that target the ubiquitously expressed prohibitins and thereby affect cellular signaling. By whole-cell patch clamp analyses, it was demonstrated that nanomolar concentrations of flavaglines increases TRPM6 activity by ∼2 fold. The stimulatory effects were dependent on the presence of the alpha-kinase domain of TRPM6, but did not require its phosphotransferase activity. Interestingly, it was observed that two natural occurring TRPM6 mutants with impaired insulin-sensitivity, TRPM6-p.Val1393Ile and TRPM6-p.Lys1584Glu, are not sensitive to flavagline stimulation. In conclusion, we have identified flavaglines as potent activators of TRPM6 activity. Our results suggest that flavaglines stimulate TRPM6 via the insulin receptor signaling pathway.

## Introduction

Magnesium (Mg^2+^) is an essential electrolyte for cell growth, protein synthesis and enzymatic activity. Therefore, physiological mechanisms maintain blood Mg^2+^ concentrations within a tightly regulated range (0.7–1.1 mM) [1,2]. The apically expressed *Transient Receptor Potential Melastatin type 6* (TRPM6) channels are the gatekeepers of epithelial Mg^2+^ transport in colon and in the distal convoluted tubule segment (DCT) of the kidney nephron [3]. Loss-of-function mutations of TRPM6 cause intestinal Mg^2+^ malabsorption and renal Mg^2+^ wasting, as evidenced in patients suffering from hypomagnesemia with secondary hypocalcemia (HSH, OMIM #602014) [4,5].

TRPM6 channels are thought to form tetramers of subunits comprising six transmembrane segments, with a central divalent-selective pore (Ba^2+^>Ni^2+^>Mg^2+^>Ca^2+^) [3]. Functional channels are inhibited by intracellular Mg^2+^ [3,6] and consequently display a time-dependent increase in currents upon dialysis of cells with a pipette solution containing a strong Mg^2+^ chelator such as ethylenediaminetetraacetic acid (EDTA). Like its close homolog TRPM7, TRPM6 channels comprise an intrinsic intracellular Ser/Thr kinase domain, which has similarities to proteins of the alpha-kinase family [7]. TRPM6 channels undergo autophosphorylation, but the role of the alpha-kinase on channel function and cell physiology is still incompletely understood [6,8–11].

Over the last decade, the epidermal growth factor (EGF) and insulin were shown to stimulate the activity and membrane expression of TRPM6 [12,13]. Two TRPM6 single nucleotide polymorphisms (SNPs: p.Val1393Ile and p.Lys1584Glu) were recently associated with an increased risk of diabetes development in humans [13,14]. Subsequently, it was shown that these mutations prevent a rapid insulin-evoked increase in channel plasma membrane expression [13].

By combined pull down and mass spectrometry studies of the TRPM6 alpha-kinase domain, three interacting proteins have been identified: I) *Methionine sulfoxide reductase B1* (MSRB1) which reduces the sensitivity of TRPM6 to oxidative stress [15], II) *Guanine nucleotide-binding protein subunit beta-2-like 1* (GNB2L1/RACK1) which inhibits TRPM6 activity in a alpha-kinase-dependent manner [16]. III) *Prohibitin 2* or *Repressor protein of Estrogen receptor Activity* (PHB2/REA) which inhibits TRPM6, an effect that is relieved by estrogens [17].

Prohibitins (PHB1 and PHB2) are ubiquitously expressed members of the family of stomatin/prohibitin/flotillin and HflK/C (SPFH) domain containing proteins [18–20]. PHBs are found in the nucleus, cytoplasm and plasma membrane, where they play an important role in cellular differentiation, anti-proliferation and mitochondrial morphogenesis. PHBs modulate the cell cycle progression, regulate transcription and facilitate cell surface signaling [18,19]. Recently, a family of natural compounds named flavaglines was established as high affinity ligand of PHBs [21].

Flavaglines are a family of natural compounds characterized by a cyclopenta*[b]*benzofuran structure [22]. Natural flavaglines and synthetic analogs have been intensively studied, owing to their pleiotropic favorable properties (anti-inflammatory, anticancer, cardioprotective and neuroprotective) [23]. Flavaglines bind PHB1 and PHB2 (with nM affinity) and prevent the CRaf-mediated activation of oncogenic MAPK signaling [21]. Additionally and independently from PHBs, flavaglines inhibit *eukaryotic initiation factor-4A* (eIF4A)-dependent oncogenic protein synthesis [23]. In addition, the binding properties of flavaglines to PHB and/or eIF4A lead to the induction of apoptosis in *apoptosis inducing factor* (AIF) and caspase-12-dependent manners [23,24]. The mechanism of flavaglines neuro- and cardioprotection are likely mediated by their PHB-interacting properties, thereby reducing oxidative stress, deleterious growth factor signaling and release of inflammatory mediators [23].

Given the previously described inhibitory interaction of PHB2 on TRPM6 and the high affinity binding of flavaglines to PHB1 and PHB2, this study aims to identify and characterize the effect of flavaglines on TRPM6 activity.

## Materials and Methods

### Cell culture

Human embryonic kidney cells (HEK293) were grown at 37°C in DMEM (Biowhittaker Europe, Vervier, Belgium) supplemented with 10% (v/v) fetal calf serum (PAA Laboratories, Linz, Austria), non-essential amino acids and 2 mM L-glutamine in a humidified 5% (v/v) CO_2_ atmosphere. Cells were seeded in 12-well plates and subsequently transfected with 1 μg of human NH_2_-terminal HA-tagged TRPM6 or empty pCINeo IRES GFP vectors (mock) cDNA using Lipofectamine 2000 (Invitrogen) at 1:3 DNA:Lipofectamine ratio. For patch clamp experiments, cells were seeded two days after transfection on glass coverslips coated with 50 μl/cm^2^ of 50 μg/ml fibronectin (Roche, Mannheim, Germany). Two hours later, cells were placed in the recording chamber and selected based on the intensity of the fluorescent reporter.

### Electrophysiology

All experiments were undertaken and analyzed using an EPC-9 amplifier and the Patchmaster software (HEKA electronics, Lambrecht, Germany). The sampling interval was set to 200 ms and data was low-pass filtered at 2.9 kHz. Patch clamp pipettes were pulled from thin-walled borosilicate glass (Harvard Apparatus, March-Hugstetten, Germany) and had resistance between 1 and 3 MΩ when filled with the pipette solution. Series resistance compensation was set to 75–95% in all experiments. Current densities were obtained by normalizing the current amplitude to the cell capacitance.

### Compound synthesis and purity

FL2, FL3 and FL23 were synthesized as previously described [24,25]. Purity of the compounds was >95%, as assessed by reversed-phase high performance liquid chromatography (HPLC) analyses (Hypersil Gold column 30×1 mm, C18, Thermo Scientific) under the following conditions: flow rate: 0.3 mL/min; buffer A: CH_3_CN, buffer B: 0.01% aqueous Trifluoroacetic Acid (TFA); gradient: 98–10% (v/v) buffer B over 8 min (detection: λ = 220/254 nm).

### Solutions and compound application

The extracellular solution contained (in mM): 150 NaCl, 1 CaCl_2_, 10 HEPES/NaOH pH 7.4. The pipette solution was made of (in mM): 150 NaCl, 10 Na_2_EDTA, 10 HEPES/NaOH pH 7.2 [3]. Cells were pre-incubated 15 minutes at 37°C in bath solution containing the compound of interest diluted from a 1000x stock solution or vehicle (0.1% v/v dimethyl sulfoxide (DMSO)).

### Immunoblotting

HEK293 cells were lysed for 1 hour at 4° C in TNE lysis buffer containing (in mM): 50 Tris/HCl (pH 8.0), 150 NaCl, 5 EDTA, 1% (v/v) Triton X-100 and protease inhibitors (pepstatin 1 μg/ml, PMSF 1 mM, leupeptin 5 μg/ml and aproptin 5 μg/ml). Protein lysates were denatured in Laemmli containing 100 mM dithiothreitol (DTT, 30 minutes, 37°C) and subsequently subjected to SDS-PAGE. Immunoblots were incubated with mouse anti-HA (Roche, high affinity 3F10, 1:5,000), rabbit anti-Akt (Cell signaling, 1:1000) and rabbit anti-ERK1/2 (Cell signaling, 1:1,000) primary antibodies and peroxidase conjugated sheep anti-mouse secondary antibodies (Jackson Immunoresearch, 1:10,000).

### Statistical analysis

All results are depicted as mean ± standard error of the mean (SEM). Statistical analysis was conducted by one-way Student’s t-test when comparing two treatment groups or experimental conditions. Difference in means with P values <0.05 were considered statistically significant and indicated by a star (*).

### Curve fitting

Current time-development curves were fitted with a logistic equation: I = I_0_+((I_max_-I_0_)/(1+(t/t_1/2_)^-h^)^s^), with I the current density, I_0_ the baseline current density, t the time, t_1/2_ the time of half-maximal current density, h the slope and s a parameter. Half-maximal stimulatory concentration (EC_50_) was obtained by fitting a Hill equation to the data points: I = I_0_+(I_max_—I_0_) *(([FL23]^n^)/(IC_50_^n^+[FL23]^n^)), with I the current density, I_0_ the baseline current density obtained in control conditions, I_max_ the maximal current value and n the Hill equation.

## Results

### Synthetic flavaglines stimulate TRPM6 activity

**Fig 1:**
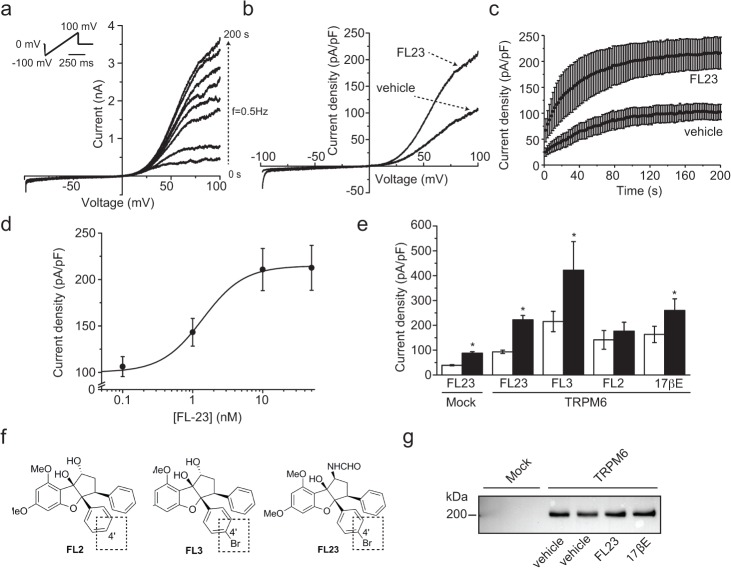
Flavaglines stimulate TRPM6 at nanomolar concentration. **a.** TRPM6 currents were evoked by a series of 500 ms voltage ramp from -100 to +100 mV applied every 2 s (0.5 Hz) from a holding potential of 0 mV (top left inset). A typical set of current-voltage curves obtained from a single cell is shown. **b.** Typical current-voltage curves obtained 200 s after break-in from cells pre-incubated 15 minutes with vehicle or FL23 (50 nM). **c.** The average time-course of TRPM6 current development with (n = 19) or without (n = 19) FL23 pre-treatment are shown for current values measured at +80 mV. **d.** FL23 increases TRPM6 current density in a concentration-dependent manner (n≥3 per data points). Line represents the fit of data points with a Hill equation (see Material and Methods). **e.** Incubation of TRPM6 expressing cells with FL3 (50 nM, n≥10), FL23 (50 nM, n≥22) or 17βE (50 nM, n≥9) significantly increased the average current densities measured at +80 mV 200 s after break-in. Mock-transfected cells showed a similar increase in current density (n≥8). TRPM6 currents were not sensitive to FL2 (n≥10). Stars indicate statistically significant difference (P<0.05) between vehicle- (white bar) and compound-treated cells (black bar). **f.** The chemical structures of FL2, FL3 and FL23 are shown. **g**. Cells were pre-incubated with vehicle, FL23 (50 nM) or 17βE (50 nM). Total lysate were subjected to Western blot analysis using an anti-HA primary antibody. FL23 and estradiol (17βE) did not significantly affect the expression of TRPM6. A representative blot of three separate experiments is shown.

**Fig 2:**
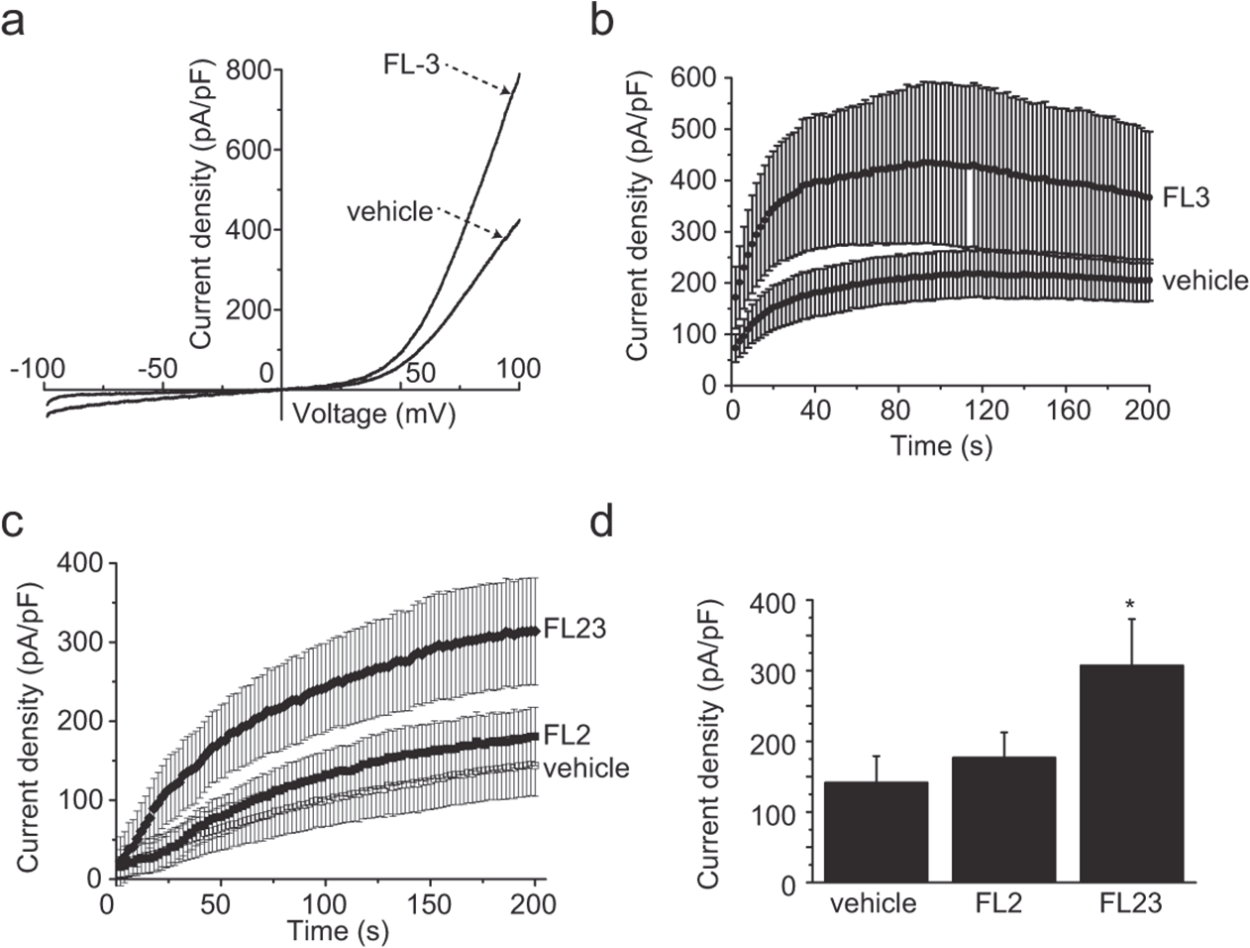
Analogs of FL23 show distinct effects on TRPM6 currents. **a.** Typical current-voltage curves obtained 200 s after break-in are shown for vehicle and FL23 (50 nM) pre-incubated cells. **b.** The average time-course of TRPM6 current development with (n = 8) or without (n = 11) FL23 (50 nM) is shown for current values measured at +80 mV. **c.** The average time-course of TRPM6 current development with FL2 (50 nM, n = 12), vehicle (n = 10) or FL23 (50 nM, n = 10) pre-treatment is shown for current values measured at +80 mV. **d.** FL2 incubation did not significantly stimulate TRPM6 activity (n≥10). Stars indicate statistically significant difference (P<0.05) between vehicle and compound-treated cells.

HEK293 cells were transfected with the previously described pCINeo-TRPM6-HA-IRES-GFP vector [3]. This construct allows the visual identification of cells expressing TRPM6. Cells were then subjected to whole-cell patch clamp analysis, as previously described [3]. Briefly, currents were elicited by a series of voltage ramps applied at 0.5 Hz from a holding voltage of 0 mV. Due to the dialysis of the cytoplasm with a pipette solution containing EDTA, time-dependent outwardly rectifying currents were observed in response to this ramp protocol (Fig. 1A). In order to assess the effect of flavaglines on TRPM6, cells were first exposed to FL23 (50 nM) [25], a potent analog of the established PHB2 ligand FL3 [21]. This protocol yielded a significant increase in the current density without affecting the characteristic shape of the current-voltage (IV) curve (Fig. 1B) or the current time-development characteristics (Fig. 1C). The average time-development curves were fitted with a logistic equation (see Material and Methods). This analysis revealed that the time of half-maximal activation (t_1/2_) was not changed between control and FL23-treated cells (control: 48 ± 2 s, FL23: 43 ± 3 s). The rate of current development (the slope *h*) was increased with FL23 treatment (control: 1.8 ± 0.1 pApF^-1^s^-1^, FL23: 3.0 ± 0.2 pApF^-1^s^-1^). Pre-incubation of the cells with concentrations of FL23 ranging from 0.01 to 50 nM revealed a concentration-dependent stimulation of TRPM6 activity with an EC_50_ = 1.4 ± 0.2 nM and Hill equation n = 1.5 ± 0.3 (see Material and Methods, Fig. 1D). A similar increase in TRPM6 activity was observed with FL3 (50 nM, Fig. 2A-B). Next, cells were pre-incubated with FL2 (50 nM, Fig. 1F), a flavagline that does not display significant cytotoxicity in cancer cells [24] nor cytoprotection in cardiomyocytes [26]. In contrast to FL3 and FL23, this treatment did not significantly alter the current density of TRPM6-expressing cells (Fig. 2C-D). As previously reported, estradiol (17βE) significantly stimulated TRPM6 currents (Fig. 1E) [17]. On average, 17βE, FL3 and FL23 stimulated TRPM6 activity by 1.5 to 2-fold (Fig. 1E). Interestingly, mock-transfected cells demonstrated a similar ∼2-fold increase in current density upon FL23 treatment, indicating that TRPM7 is also a likely target of flavaglines action (Fig. 1E). As expected from the short pre-incubation period, the expression of TRPM6 was not influenced by FL23 or 17βE (Fig. 1G).

### The stimulating effects of flavaglines require the intrinsic kinase domain of TRPM6

**Fig 3:**
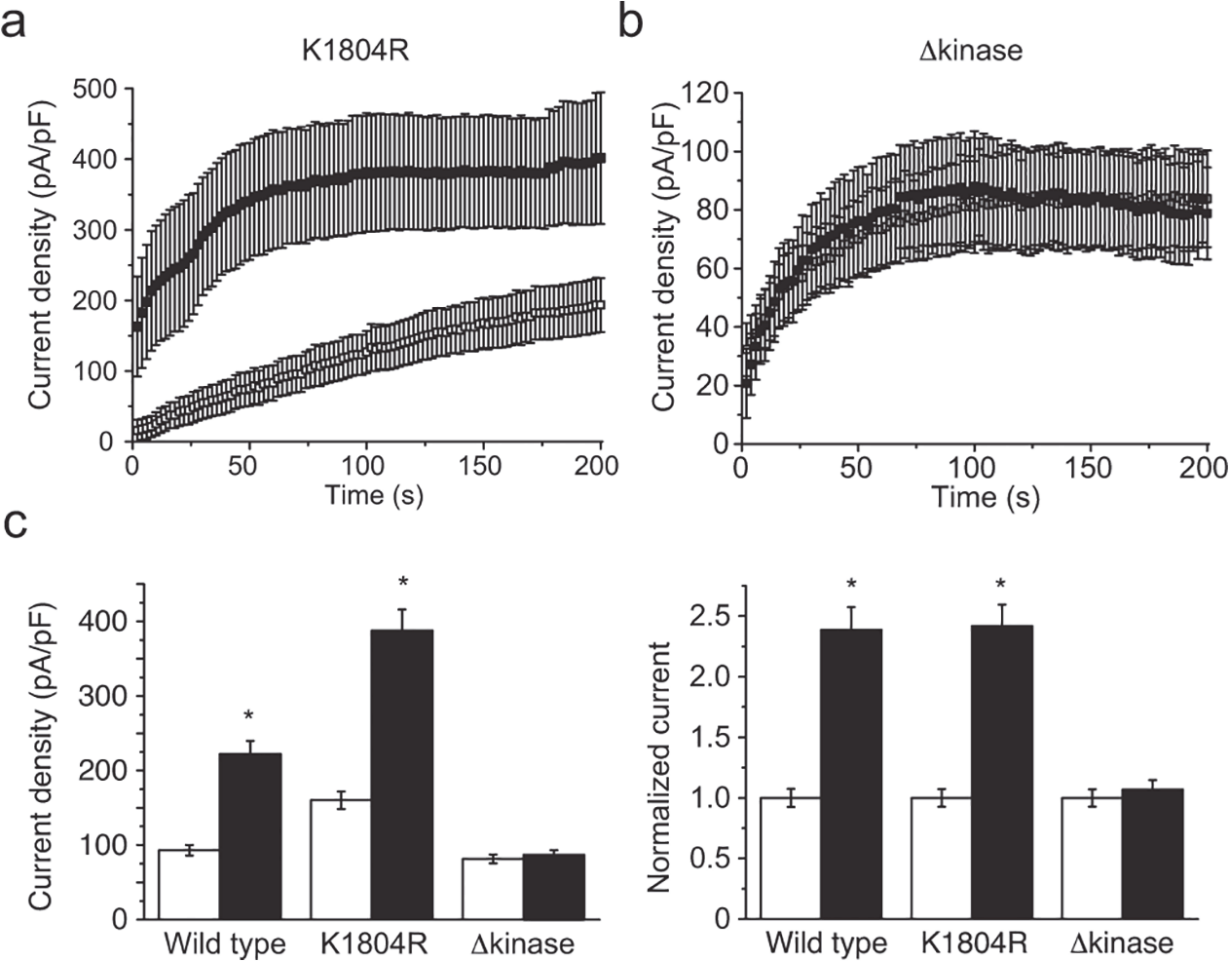
The presence of the intrinsic alpha-kinase domain of the channel but not its activity is required for flavagline-mediated stimulation of TRPM6. **a.** The average time-course of current development of the kinase-inactive channels (TRPM6^K1804R^) with (n = 7, full symbols) or without (n = 9, empty symbols) FL23 (50 nM) pre-incubation is shown for current values measured at +80 mV. **b.** The average time-course of current development of TRPM6^L1749X^ (Δkinase) with (n = 8, full symbols) or without (n = 9, empty symbols) FL23 (50 nM) pre-incubation is shown for current values measured at +80 mV. **c.** Pre-incubation of cells with FL23 (50 nM) stimulated wild type (n≥22), K1804R (n≥9), but not Δkinase (n≥8, p>0.05) channel activity. Right panel shows current values normalized to each control condition. Stars indicate statistically significant difference (P<0.05) between vehicle- (white bar) and compound-treated cells (black bar).

To assess the involvement of the intrinsic alpha-kinase domain in the flavagline-mediated potentiation of TRPM6 currents, cells were transfected with the previously described kinase-truncated (p.Leu1749*, Δkinase) or kinase-inactive (p.Lys1804Arg, KI) TRPM6 constructs [9]. While the first construct produces mutant channels lacking the complete kinase domain, the KI construct form channels without intrinsic alpha-kinase phosphotransferase activity. Both constructs produce functional proteins with apparently normal channel function in the absence of intracellular Mg^2+^. Using an identical pre-incubation protocol, cells expressing the KI mutant demonstrated a FL23-mediated increase in current densities similar to wild type (Fig. 3A and C). In contrast, the Δkinase mutant failed to respond to this treatment (Fig. 3B-C).

### Flavaglines act along a shared pathway with insulin

**Fig 4:**
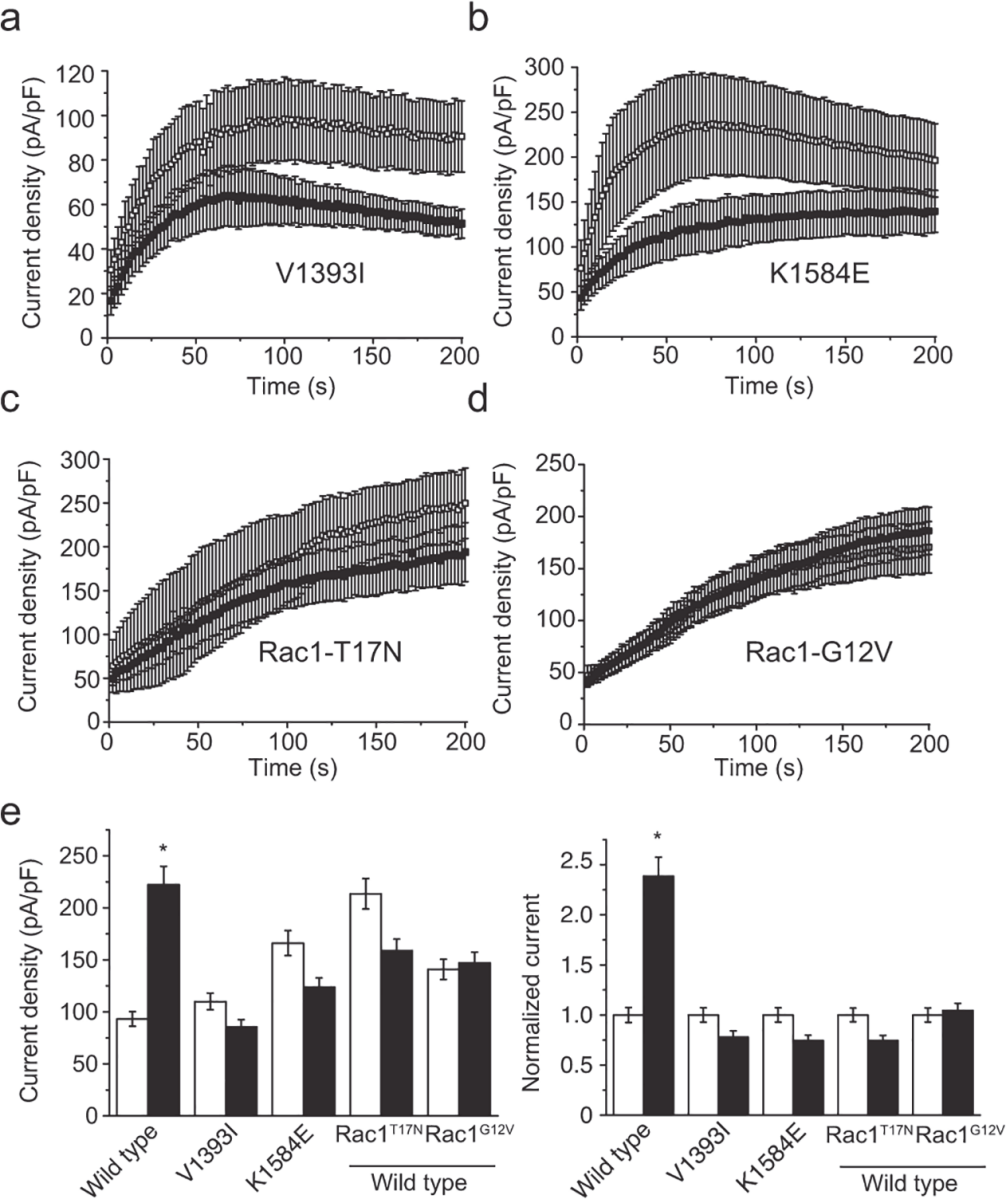
Flavaglines act upon a common pathway with insulin receptor signaling. **a-d.** The average time-course of current development of cells pre-incubated with (full symbols) or without (empty symbols) FL23 (50 nM) for: (**a**) TRPM6^V1393I^ (n≥8), (**b**) TRPM6^K1584E^ (n≥7), (**c**) wild type TRPM6 together with Rac1^T17N^ (n≥6) and (**d**) wild type TRPM6 together with Rac1^G12V^ (n≥11). **e.** FL23 pre-incubation failed to alter currents in cells expressing TRPM6^V1393I^ (n = 11), TRPM6^K1584E^ (n≥8) and cells co-expressing wild type TRPM6 together with Rac1^T17N^ (n = 8) and TRPM6 together with Rac1^G12V^ (n≥14). Right panel shows current values normalized to each control condition. Stars indicate statistically significant difference (P<0.05) between vehicle- (white bar) and compound-treated cells (black bar).

The intracellular amino acid residues p.Val1393 and p.Lys1584 have been shown to independently confer sensitivity of TRPM6 channels to insulin stimulation, probably by altering the phosphorylation of the neighboring p.Thr1391 and p.Ser1583 residues, respectively [13]. Phosphomimicking mutations of either p.Thr1391Asp or p.Ser1583Asp were shown to be permissive in the insulin-mediated potentiation of TRPM6 [13]. To address whether flavaglines act on TRPM6 in a similar way as insulin, cells expressing either of two naturally occurring insulin-insensitive TRPM6 SNPs (p.Val1393Ile or p.Lys1584Glu) were pre-incubated with FL23 (50 nM). These mutants failed to respond to FL23 (Fig. 4A-B and E).

Following the activation of the insulin receptor, a complex multi-branched signaling cascade is activated. One of these branches involves the activation of *Phosphoinositide 3-kinase* (PI3K), Akt and *Ras-related C3 botulinum toxin substrate 1* (Rac1) [27]. Co-expression of TRPM6 together with the constitutively active (p.Gly12Val) or dominant-negative (p.Thr17Asn) mutants of Rac1 have been shown to respectively allow and prevent the increase of TRPM6 membrane expression by insulin [13]. Here, cells were co-transfected with TRPM6 and either the p.Thr17Asn or p.Gly12Val Rac1 mutants. Both mutants prevented the stimulation of TRPM6 by FL23 (Fig. 4C-D and F).

### Flavaglines do not affect Akt phosphorylation

**Fig 5:**
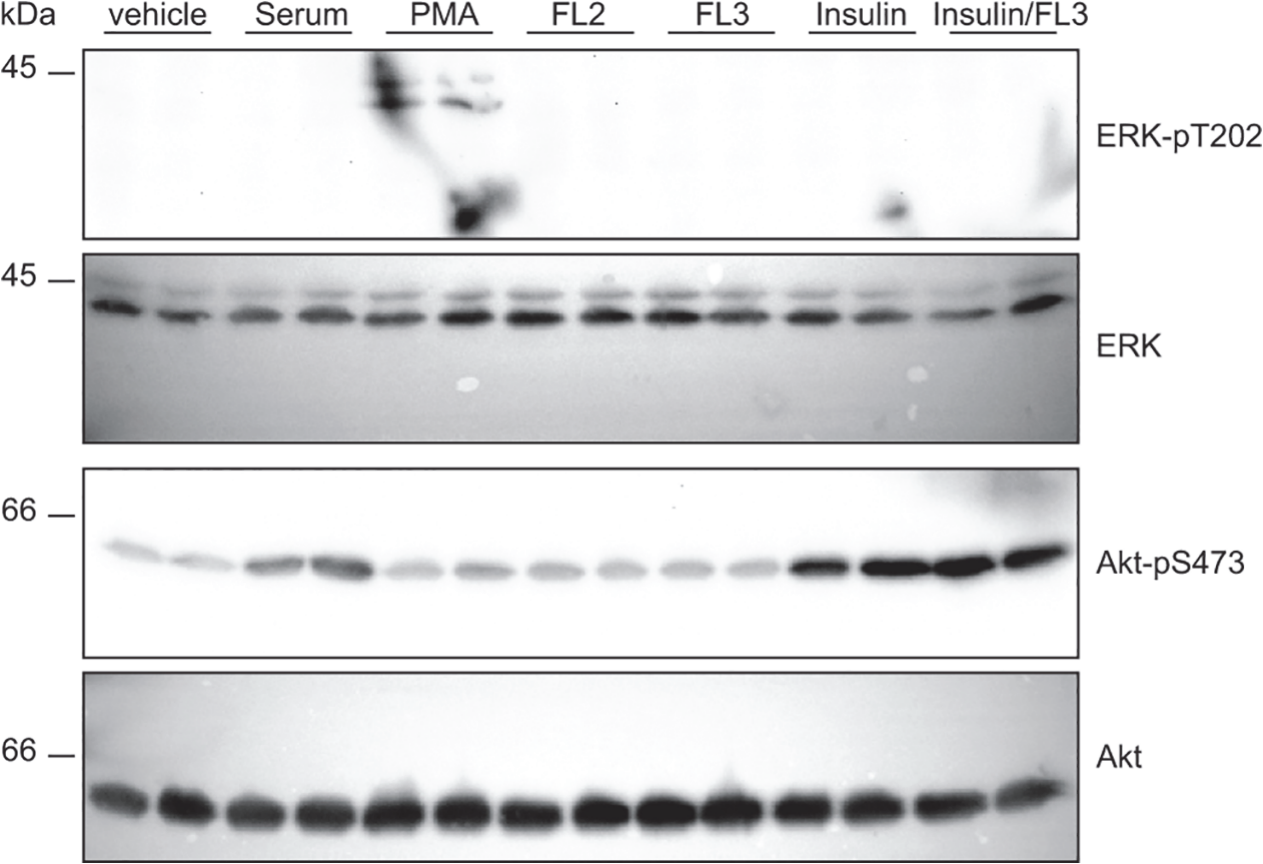
Cellular Akt and ERK signaling is unaffected by FL3. HEK293 cells were incubated with FL2 (50 nM), FL3 (50 nM), PMA (100 nM), insulin (10 nM) or 17βE (50 nM) for 15 minutes. Protein lysates were immediately obtained and immunoblots were performed to detect pERK1/2 and pAkt. PMA and insulin served as positive controls for ERK and Akt phosphorylation, respectively.

Given the previously described modulation of Akt by PHBs [28], the effects of flavaglines on Akt phosphorylation were examined using the same experimental conditions as were used in the patch clamp experiments. Following 15 minutes of incubation, phosphorylation of Akt was not induced by FL2, FL3 and FL23 (50 nM, Fig. 5). It has been previously demonstrated that flavaglines prevent ERK1/2 phosphorylation in a manner that depends on CRaf [21]. Here, basal ERK1/2 phosphorylation was not apparent in control condition and no additional phosphorylation was detected upon flavaglines stimulation (Fig. 5).

## Discussion

The present study demonstrates that the activity of the Mg^2+^-permeant TRPM6 channel is stimulated ∼2-fold by the flavaglines compounds FL3 and FL23. This is the first report of an exogenous natural compound that stimulates TRPM6 activity.

**Fig 6:**
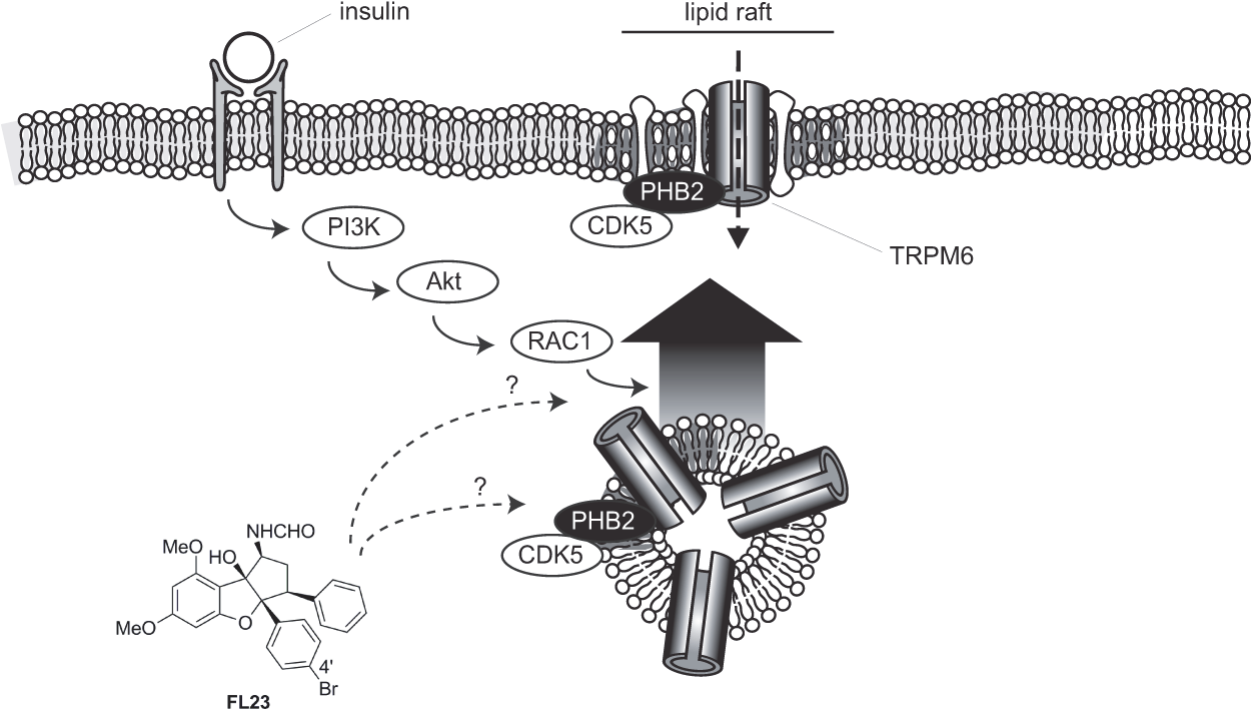
Proposed model of flavaglines action. Flavaglines stimulate TRPM6 activity by acting on downstream effector(s) of the insulin receptor. PHB2 and CDK5, proteins which are known to regulate TRPM6 are localized in lipid rafts.

The activity of TRPM6 and its plasma membrane expression have been shown to be increased upon stimulation with insulin. This effect relied on the PI3K, Akt and Rac1 signaling cascade (Fig. 6). Detailed electrophysiological and total internal reflection fluorescence (TIRF) microscopy analyses have revealed a permissive role for TRPM6-p.Val1393 and TRPM6-p.Lys1584 sites in insulin-evoked insertion of channels in the plasma membrane [13]. Here, it is proposed that flavaglines stimulate TRPM6 by acting along the same pathway (Fig. 6). This hypothesis is based on the following observations: (I) flavaglines increased TRPM6 activity ∼1.5–2 fold, which is quantitatively similar to the previously described action of insulin on TRPM6 activity [13]; (II) the insulin-insensitive (TRPM6-p.Val1393Ile and TRPM6-Lys1584Glu) TRPM6 mutants were not potentiated by flavaglines; (III) flavaglines-induced TRPM6 stimulation was absent when overexpressing TRPM6 together with Rac1 mutants; (IV) in concordance with the mechanism of insulin action on TRPM6, flavaglines stimulated the kinase-inactive (TRPM6-p.Lys1804Arg) mutant. Taken together, it is hypothesized that flavaglines act by triggering or relieving a tonic inhibition on one (or more) of the molecular player(s) involved in insulin signaling, effectively promoting the plasma membrane insertion of wild type TRPM6 channels but not of TRPM6 channels containing insulin-insensitive SNP mutants. Our data suggest that the effects of flavagline-stimulation take place downstream of Akt, since no additional Akt phosphorylation was evident upon treatment with FL3. Further experiments investigating the detailed molecular action of flavaglines on the localization and phosphorylation of the known kinases involved in growth-factor stimulation of TRPM6 (PI3K/Akt/Rac1/Cdk5) will be necessary to elucidate the exact molecular targets.

Flavaglines have recently been identified as potent interactors of PHBs [18,19]. Interestingly, PHB1 and PHB2 are enriched in detergent resistant (lipid rafts) fractions of the plasma membrane [18]. Given the previously described action of PHBs as chaperone of Ras-dependent CRaf activation in the plasma membrane [21], a general function of PHBs is to provide spatial constraints necessary for the proper regulation of proteins in specialized regions (*eg* the lipid rafts) of the plasma membrane. It can be hypothesized that TRPM6 channels transiently or permanently localize together with PHB in the lipid raft fractions of the plasma/vesicular membrane, where channels undergo regulatory phosphorylation. The following facts support this hypothesis: (I) TRPM6 has been shown to establish a inhibitory interaction with PHB2 [17]; (II) TRPM6 requires CDK5 phosphorylation for proper insulin-mediated regulation, CDK5 localizing and being activated in the lipid raft fraction of plasma membrane [29]; (III) the close homolog TRPM7 has been reported to localize in lipid rafts [30], (IV) TRPM6 requires PIP2 for proper function [31], a lipid that is enriched in lipid rafts. Therefore, it is tempting to speculate that PHBs binding to TRPM6 promotes the formation of a macromolecular regulatory complex in the lipid rafts of the plasma membrane. In this perspective, it is interesting to note that the insulin-induced signaling pathway becomes more active when the insulin receptor is expressed in lipid rafts [32–34]. In addition to the inhibitory PHB2-TRPM6 interaction, other TRP channels are modulated by members of the SPFH protein family [20]. Podocin, an SPFH protein similar to prohibitin, regulates the insulin sensitive *transient receptor potential canonical type 6* (TRPC6) ion channel in the kidney [35]. In line with the current hypothesis, it has been proposed that podocin organize TRPC6-lipid complexes in the plasma membrane, thereby modulating channel activity [36]. Altogether, these findings point towards a compartmentalized insulin signaling cascade on/near the lipid rafts in the vesicular/plasma membrane. In this model, expression of TRPM6 and the insulin receptor in the lipid rafts allows for the rapid local regulation of TRPM6 by insulin.

Comparison of structure-function activity between active (FL3, FL23) and inactive (FL2) flavaglines analogs revealed a positive correlation between cytostatic/cytotoxic properties of flavaglines in cancer cell lines and their action on TRPM6 [21,24,25]. While the effects of flavaglines on cellular proliferation and growth factor signaling were evident starting from 2 hours after compound application, the stimulatory effect shown here occurs within 15 minutes. These results suggest that the short-term effects of flavaglines on TRPM6 take place independently from any translational effect (*eg* eIF4A-dependent). However, the concurrent structure-function relationship of flavaglines in their TRPM6-stimulatory effects and in their cytotoxic properties suggests that both mechanisms are PHB-dependent. It has previously been shown that PHB2 interacts with TRPM6 and that 17βE reduces this inhibitory interaction [17]. However, the current results suggest that 17βE and FL23 only share a partially overlapping mechanism of action. Exogenous PHB2 inhibits TRPM6 in an alpha-kinase phosphotransferase-dependent manner [17]. In contrast, flavaglines stimulated the kinase-inactive TRPM6-p.Lys1804Arg mutant. Moreover, flavaglines induce an increase in endogenous TRPM7 currents, while TRPM7 currents were insensitive to PHB2-mediated inhibition [17]. Further work identifying the major players that are part of the TRPM6-PHB macromolecular complex and its regulation by flavaglines and 17βE are necessary to further understand the stimulatory but slightly distinct effects of these compounds on the activity of TRPM6.

Evidences suggest that the stimulatory action of flavaglines is not restricted to TRPM6. Here, a stimulatory effect of FL23 was also observed in mock-transfected cells. Given the experimental conditions used in this study, a substantial part of the current in mock-transfected HEK293 cells is carried by endogenous TRPM7 channels [3]. Further experiments investigating the action of flavaglines on other members of the SPFH protein and TRP channel (*eg* TRPC6) families will be needed to understand the complex mechanism of action of flavaglines.

Reduced TRPM6 channel activity results in a clinically relevant hypomagnesemia due to renal Mg^2+^ wasting. Because insulin and EGF stimulate TRPM6 function, patients with diabetes mellitus type 2 or users of EGFR inhibitors are at risk to develop hypomagnesemia [12,13]. Given that FL3 and FL23 stimulate TRPM6 activity, flavaglines may provide an important therapeutic potential for these patient groups. A preliminary experiment with FL3 (daily i.p. injection 0.1 mg/kg, 7 days,) did not reveal changes in serum or urinary Mg^2+^ concentration in mice (data not shown). In addition to the optimization of treatment duration, the actual dose of FL3 reaching the DCT cells in the kidney where TRPM6 is located may be much lower and challenging to assess. Future experiments assessing the bioavailability of flavaglines should be performed to assess putative magnesiotropic effects *in vivo*. Additionally, given the physiological mechanism of intestinal and/or renal compensation of Mg^2+^ transport, the effects of flavaglines should be assessed in a murine model of hypomagnesemia.

In conclusion, the natural compounds flavaglines stimulate the activity of TRPM6 Mg^2+^ channels at nanomolar concentrations. The effect is rapid (within 15 minutes) and probably relies on a near-plasma membrane mechanism that likely involves PHBs.
